# PP76 “It Is Better For Me To Die Than To Be Disgraced”: Perceptions Of Worse-Than-Death Health States In Ghana

**DOI:** 10.1017/S0266462324002459

**Published:** 2025-01-07

**Authors:** Adwoa Essel, Patricia Akweongo, Rebecca Addo, Justice Nonvignon, Akosua Adjeiwa Affram

## Abstract

**Introduction:**

Many cultures across the world have varying conceptions about death and dying. Perceptions about health states considered “worse than death” also vary based on sociocultural norms as well as health system capacity. We explore worse-than-death health states in Ghana as well as reasons for opting for death in those health states.

**Methods:**

We interviewed 28 participants from three regions in Ghana to understand the contextual “value of life” in Ghana and factors influencing respondents’ decision to opt to die rather than live in a particular health state. Written consents were sought from all participants to partake in the study. Interviews were conducted in either Twi or English based on each participant’s preference and lasted for an average of 30 to 35 minutes. Interviews were transcribed verbatim and stored in NVivo software. Data were analyzed thematically.

**Results:**

We identified that health states perceived as worse than death were those associated with impairment in mobility, anxiety/depression, and pain/discomfort. Participants preferred death under these circumstances because they wanted to avoid the financial burden on themselves and family, time spent in caregiving by family, loss of personhood, and loss of social status. Decisions regarding health states worse than death hold considerable importance, particularly in a context where culture and societal norms play a role in shaping how quality of life is assessed.

**Conclusions:**

An understanding of the value Ghanaians attach to health states perceived as worse than death provides useful information for patient-centered care. Findings from the study can provide evidence on healthcare resource allocation and aid policymakers and clinicians in making informed decisions on which treatments to prioritize, and how to maximize the overall health and well-being of individuals.

